# The history of the cardiac emergency room, and the patient’s history

**DOI:** 10.1007/s12471-014-0625-x

**Published:** 2014-11-07

**Authors:** R. J. G. Peters

**Affiliations:** Department of Cardiology, F3-236, Academic Medical Center, PO Box 22660, 1100 DD Amsterdam, the Netherlands

The first Cardiac Emergency Room (CER) was founded in 1978 by Professor Dirk Durrer in Amsterdam. CERs have since then been opened in many countries, with the initial goal of reducing time delays in patients with acute myocardial infarction. In this role they have contributed to the reductions in mortality from coronary disease that have been observed in the past decades [1]. In later years, the focus of CERs has gradually shifted towards ruling out acute coronary syndromes (ACS), to reduce unnecessary hospital admissions [2].

In more recent years, other clinical conditions have been added to the case loads of CERs, including worsening heart failure, atrial fibrillation and syncope. This is the consequence of, among other things, increasing numbers of patients who have survived a previous cardiac event, of reduced capacity at outpatient clinics, of uncertainty among general practitioners and of lower thresholds for self-referral by well-informed patients. As a result, CERs in many centres are receiving growing numbers of patients. Daily challenges are therefore increasingly logistic in nature. One of the undesirable effects is that the goals of CER staff may shift from understanding a patient’s complaints and providing appropriate care to early discharge and the creation of vacancy.

The study by Willems et al., published in this issue [3], was performed against this background. The authors investigated a new rule-out protocol that was aimed at early discharge of patients presenting with chest pain who are at low risk of serious adverse events. This risk was assessed by a number of clinical characteristics (combined in the ‘Heart Score’), and by ruling out myocardial damage as measured by ‘high sensitive’ troponin T in plasma, at three time points after the onset of symptoms.

As expected, both clinical parameters of risk and evidence of myocardial damage were associated with increased risk of adverse events in the first month following discharge from the CER. Similarly expected, ‘high sensitive’ troponin T had higher sensitivity for detection of myocardial damage than the conventional troponin T assay (both by Roche Diagnostics). In patients who were classified as low risk by both clinical and biochemical characteristics, early discharge with outpatient clinical follow-up appeared to be safe. This is consistent with previous studies on comparable approaches [4]. Thus, the approach described in this paper may assist clinical decision making and support effective use of the CER, as the authors suggest. In most CERs high sensitive troponin has now replaced earlier assays of myocardial damage. The findings of the current and similar studies may therefore be widely used to guide clinical practice.

The quality of the current study is mainly limited by the low number of patients that were included. For a reliable estimate of the clinical outcome of major adverse cardiac events, the observation would need to be about 100-fold larger than the 89 patients in the present study. Second, one of the most important components of the clinical assessment at the CER is the nature of the presenting complaint. ‘Chest pain’ is an ambiguous qualification of the patients’ presenting symptoms. The nature of the presenting complaint is included in the assessment in the current study, as one of the components of the ‘Heart Score’. However, it is reduced to a value of zero, 1 or 2 depending on how ‘typical’ the symptoms are. Careful history taking by an experienced clinician will likely classify many patients correctly by history alone, both in diagnostic and prognostic terms. This is supported by good evidence in patients with stable chest pain. Little evidence is available on patients presenting with chest pain at rest, and it is clear that history alone is not sufficient to classify a patient’s risk. Importantly, up to 20 % of patients with ACS have atypical chest pain and a nondiagnostic electrocardiogram [5]. Still, most seasoned clinicians would agree that diagnostic testing cannot replace a careful history. In addition, without an understanding of clinical presentation the interpretation of the results of laboratory tests may lead to serious error. It is unfortunate, therefore, that recently published decision trees start from the artificially uniform entry term of ‘chest pain’ [6]. Third, drug therapy after discharge or after admission was not reported in the study. Clearly, with appropriate medication the risk of adverse events during follow-up will be lower than with insufficient medication, both after discharge and after admission. Finally, noninvasive imaging or functional (stress) testing is not included in the present study. Stress testing is safe, with careful patient selection, and provides valuable information to support the management of patients who present with chest pain [7]. The potential value of recent imaging modalities has been demonstrated and needs to be considered when designing new rule-out protocols [8].
